# How does educational inequality affect residents’ subjective well-being?—Evidence from China

**DOI:** 10.3389/fpsyg.2024.1432789

**Published:** 2024-11-28

**Authors:** Difeng Lin, Zeyun Liu

**Affiliations:** School of Economics and Management, Beijing Normal University, Beijing, China

**Keywords:** residents’ well-being, educational inequality, income disparity, economic growth, econometric analysis

## Abstract

In the context of promoting educational equity and improving welfare, exploring ways to further enhance residents’ subjective well-being from the perspective of educational equity holds significant practical importance. This study uses the educational Gini coefficient to measure the educational inequality index across different provinces and cities, and matches it with data from the China Family Panel Studies (CFPS) to investigate the relationship between educational inequality and residents’ subjective well-being. The research findings reveal a significant negative correlation between educational inequality and residents’ well-being, with observed heterogeneity. Specifically, educational inequality has a greater negative impact on groups with lower levels of well-being, rural areas, and the central and western regions of China. Mechanism analysis confirms the income distribution effect and economic growth effect of educational inequality. Therefore, increasing attention to the issue of educational equity and understanding the well-being effects of educational inequality are of great significance for the Chinese government in improving residents’ welfare in the new era.

## 1 Introduction

Subjective well-being refers to the sense of pleasure individuals experience when subjectively evaluating their quality of life and the value of their existence. It not only influences individuals’ physical and mental health but also serves as a crucial indicator for measuring a nation’s level of welfare. As nations focus on economic growth, equal attention must be given to improving the well-being and quality of life for their citizens (Nelson, 1959; Emmons, 2003). However, despite the continuous deepening of economic development in China and the steady growth of GDP, the overall national happiness has not increased but rather declined. According to the “World Happiness Report” released by the United Nations, although China’s happiness ranking has seen some improvement in recent years, the 2022 report indicates that China ranks only 72nd out of 146 countries and regions worldwide in terms of happiness. The level of national happiness is even lower than that of the 1990s.

In fact, the academic community has extensively and deeply explored the phenomenon of happiness not increasing alongside income growth. American economist Richard Easterlin termed this phenomenon the “Easterlin Paradox” (Clark et al., 2008; Tachibanaki, 2016; Slag et al., 2019), and scholars have sought to understand the underlying logic behind this phenomenon from various perspectives. Income inequality stands out as the most significant research perspective. However, the academic community has not yet reached a consensus regarding the relationship between income inequality and happiness. Research indicates that income inequality can harm residents’ happiness through a mechanism of “relative deprivation” (Shin, 2018; Tao et al., 2022; Osborne et al., 2019). However, some studies have also found that income inequality contributes to people developing optimistic expectations for the future, thereby increasing happiness (Graham et al., 2004; Boehm et al., 2015). Hence, some scholars propose that at different stages, income inequality may have varying effects on happiness, suggesting a curvilinear relationship between the two, known as an inverted U-shape (Yu and Wang, 2017). Therefore, solely examining income inequality may not fully explain the rationality behind the “happiness paradox.” The main reason for this uncertainty lies in the fact that income inequality is an outcome variable. Hence, subsequent scholars have gradually shifted their focus towards analyzing the causes of this outcome (Ahmad, 2017; Checchi and García-Peñalosa, 2008). It is worth noting that in China, education serves as a crucial means of accumulating human capital, playing a decisive role in determining individuals’ level of education and income. Thus, the distribution of residents’ education levels directly affects the income distribution of the entire society. Furthermore, educational equity is closely related to a nation’s welfare and output levels. Severe educational inequality can hinder social mobility, lead to social stratification, and even exacerbate inequalities across other dimensions of socio-economic conditions. Moreover, educational equity is closely linked to national welfare and productivity. Severe educational inequality can limit social mobility, deepen social stratification, and exacerbate disparities in other socio-economic dimensions. Thus, exploring how to further enhance national happiness by promoting education equity during periods of economic transformation holds significant theoretical and practical significance.

Given this background, this paper utilizes the Gini coefficient to measure the educational inequality among various provinces and cities. It then matches this data with micro-level survey data from the CFPS to empirically test the relationship between educational inequality and residents’ happiness. By constructing a mediation effects model, it further explores the mediating mechanisms through which educational inequality affects residents’ happiness. Thus, this study provides a new perspective and approach for research in the field of happiness economics and sheds light on unraveling the “Easterlin Paradox” in China.

The innovations of this paper are mainly reflected in the following aspects. First, compared to most existing studies that focus on the impact of income inequality on subjective well-being, this paper approaches the topic from the perspective of educational inequality, expanding the understanding of well-being. As a core indicator of social opportunity distribution, the impact of educational inequality on individual well-being has not been fully explored, thus offering a new perspective in this field. Second, this paper utilizes provincial-level educational inequality indicators, constructing an education Gini coefficient to measure the distribution of educational resources across regions in greater detail. By combining this with micro-level CFPS data, the paper provides a more precise empirical analysis framework, which stands in contrast to existing studies that primarily focus on macroeconomic data or income disparities. Finally, the paper explores the underlying mechanisms through which educational inequality affects well-being using a mediation effects model, specifically highlighting the role of intermediary variables such as income and economic development. This reveals how educational inequality indirectly affects residents’ well-being through multiple channels, helping to deepen the understanding of the complex relationship between educational equity and social welfare.

## 2 Theoretical analysis and research hypotheses

### 2.1 The overall relationship between educational inequality and well-being

The relative deprivation theory suggests that an individual’s sense of well-being is influenced by comparisons with others around them. If individuals perceive their situation as better than that of others, they are more likely to experience happiness (Nadler et al., 2020; Moore and Aweiss, 2003; Hoch and Loewenstein, 1991; Collins, 1996). Conversely, if they feel that their situation is worse than others’, they may experience a sense of “deprivation” and feel unhappy.

Bourdieu’s theory of social capital and cultural reproduction further deepens our understanding of educational inequality. According to Bourdieu, education is not only a site for the transmission of knowledge and skills but also an important mechanism for the reproduction of social and cultural capital (Nomaguchi and Milkie, 2020; Diener and Seligman, 2004; Mahendru et al., 2020). The education system, by maintaining and reproducing inequalities in the distribution of resources between social classes, further solidifies inequalities within the social structure. In this framework, educational inequality is not merely a difference in resource distribution but has a profound impact on the accumulation and transmission of social and cultural capital. This inequality leads to unequal opportunities, especially for individuals at the lower end of the social ladder, who find it more difficult to access high-quality educational resources, thereby limiting their social mobility and weakening their sense of well-being.

Thus, under the combined influence of relative deprivation theory and Bourdieu’s theory, educational inequality may have a negative impact on well-being. Relative deprivation theory explains the deprivation individuals experience through social comparison, while Bourdieu’s theory reveals how educational inequality deepens the differences in well-being through the accumulation and reproduction of social capital. These multiple mechanisms indicate that educational inequality not only affects individuals’ short-term well-being but also poses a threat to the long-term welfare of society as a whole.

Based on this theoretical foundation, the following research hypothesis is proposed:

Hypothesis 1: Educational inequality has a significant negative impact on residents’ well-being.

### 2.2 Mechanisms through which educational inequality affects well-being

According to the three-stage theory of social comparison, the indirect impact of educational inequality on well-being is reflected when individuals attribute the reasons for their inferiority in positional goods to educational inequality. Based on this, this paper analyzes the pathways through which educational inequality influences well-being from the perspectives of income distribution effects and economic growth effects.

Firstly, Income Distribution Effects: According to the human capital model of income disparity, in a market economy, individuals’ income levels primarily depend on the accumulation of human capital. Therefore, educational inequality affects income disparity through the pathway of “differences in human capital accumulation—differences in labor productivity—differences in occupational salaries.” Firstly, from the perspective of educational opportunities, the opportunity to access equitable educational resources is the primary prerequisite for accumulating human capital (Šlaus and Jacobs, 2011; Sima et al., 2020). However, in China, there exists a serious imbalance in the distribution of educational resources between urban and rural areas as well as among different regions. The higher social strata often utilize their resource advantages to access more educational resources and opportunities for advancement, especially in terms of access to higher education. Unequal educational opportunities can affect income distribution by influencing labor productivity and continuous training capabilities (Mincer, 1958). Secondly, from the perspective of the educational process, family background not only affects the availability of educational opportunities for children but also influences the quality of education they receive. On the one hand, high-income families tend to place more emphasis on education than low-income families and have sufficient financial resources to invest in the education sector to ensure that their children can access quality education. Morgan et al. was the first to categorize school education quality into key and non-key, academic and vocational. They found that higher family socioeconomic status significantly increased the probability of students entering key schools and academic education tracks, effectively confirming this (Zhang, 2017). On the other hand, schools with better education quality are more conducive to stimulating students’ learning motivation, promoting the improvement of their learning abilities, and consequently enhancing their academic performance (Sekreter, 2019; Zhang and Ma, 2023). Therefore, even in situations where educational resources are abundant and everyone has the opportunity to receive education, it is difficult to ensure that everyone receives education of equal quality. Educational inequalities in the process also contribute to widening income disparities. Lastly, if we ignore the positive role of individual effort in narrowing educational disparities, unequal educational outcomes resulting from unequal educational opportunities and processes will exacerbate income disparities through the pathway of “differences in labor productivity—differences in occupational salaries.” This, in turn, will further exacerbate the unequal distribution of educational opportunities and educational quality, thus forming a vicious cycle of “educational inequality—income inequality—widening educational inequality.” Given that income inequality is a key factor influencing residents’ well-being, and the majority of literature supports the view that income inequality is negatively correlated with residents’ well-being, this paper proposes the second research hypothesis as follows:

Hypothesis 2: Educational inequality widens income disparities, thereby reducing residents’ well-being.

Secondly, Economic Growth Effects: Since the emergence of the new growth theory, countries have started to pay attention to the role of human capital in economic growth. Empirical evidence also indicates that the accumulation of human capital is indeed a significant factor contributing to the differences in economic growth rates among countries. Therefore, education, as the primary means of human capital formation, is closely linked to economic growth. Early literature mostly argued that educational inequality would decrease the stock of human capital and inhibit economic growth (Aghion et al., 1999; Popkova et al., 2015). However, empirical evidence shows that the relationship between educational inequality and economic growth is not simply positive or negative. At different stages of development, the effect of educational inequality on economic growth varies. When the degree of educational inequality is high, it generally corresponds to a stage of low education levels and economic backwardness. In such circumstances, allowing some individuals to enjoy high-quality education can fully leverage the externality of higher education. This can stimulate overall productivity levels through the advancement of productive forces, thereby promoting economic growth. When the degree of educational inequality is at a moderate level, it is mainly caused by unequal distribution in basic education and higher education. At this stage, economic growth is mainly driven by industries such as manufacturing, which is the second sector. However, because the returns on higher education are lower than those on basic education, simply increasing investment in higher education to exacerbate educational inequality not only leads to a waste of educational resources but also risks causing structural unemployment. This situation occurs when the development of human capital fails to meet the talent demand of the production sectors in this stage of economic development, ultimately hindering economic growth (Storper and Scott, 2008). However, when the degree of educational inequality decreases to a lower level, educational inequality becomes beneficial for economic growth. This is because as education expands and deepens, the average level of education *per capita* generally increases. Economic development primarily relies on knowledge and technological innovation. At this stage, appropriately increasing the degree of educational inequality and increasing the proportion of the population with higher education can enhance the level of technological innovation in society, improve total factor productivity, and thereby promote economic growth (Wang et al., 2021). Shahbaz et al. confirmed the existence of a nonlinear relationship between educational inequality and economic growth when studying the situation of educational inequality in China from 1990 to 2010 (Shahbaz, 2010). They suggested that the government should adjust education policies according to different stages of development. Furthermore, since the reform and opening-up, with the continuous improvement of the education enrollment rate, China’s degree of educational inequality has been decreasing year by year, especially in developed regions in the east (Kanbur and Zhang, 2005). Based on this, it can be inferred that in the non-linear relationship between educational inequality and economic growth, educational inequality in most regions of China may have passed the stage of promoting economic growth and may have a suppressive effect on economic growth. Therefore, the third research hypothesis proposed in this article is as follows:

Hypothesis 3: Educational inequality suppresses economic growth, thereby negatively affecting the well-being of residents.

## 3 Research design

### 3.1 Baseline regression model

In this study, the dependent variable, well-being, is an ordered variable. To ensure the accuracy of the regression results, we employ an ordered probit model for regression analysis:


(1) $$\mathrm{Happines}s_{ijt}=\alpha {G^{edu}}_{jt}+\beta X_{ijt}+Y_{jt}+\mu_{j}+\mu_{t}+\varepsilon_{ijt}$$

In Equation 1, $\mathrm{Happines}s_{ijt}$ represents the subjective well-being of individual 𝑖 in province 𝑗at time 𝑡, $G_{jt}^{edu}$ represents the level of educational inequality in province 𝑗 at time 𝑡, $X_{ijt}$ represents individual characteristic variables, $Y_{jt}$ represents province characteristic variables, $\mu_{j}$ and $\mu_{t}$ represent province and time fixed effects respectively, and $\varepsilon_{ijt}$ represents the error term.

### 3.2 Transmission effects model

To effectively identify the pathways through which educational inequality influences well-being, this study follows the approach o Russ and Bansaland constructs a transmission effects model as shown in Equations 1, 3 below (Russ et al., 2017):

Step 1: Test the effect of educational inequality on the mediating variables (income inequality, economic growth):


(2) $$M_{jt}=\beta_{0}+\beta_{1}Edu_{jt}+\beta_{2}X_{ijt}+\beta_{3}Y_{jt}+\varepsilon_{ijt}$$

Step 2: Test the effect of mediating variables on well-being:


(3) $$\mathrm{Happines}s_{ijt}=\beta_{0}+\beta_{1}M_{jt}+\beta_{2}X_{ijt}+\beta_{3}Y_{jt}+\varepsilon_{ijt}$$

### 3.3 Variables and data sources

Dependent Variable: The dependent variable in this study is residents’ subjective well-being. To better reflect the “subjectivity” of residents’ happiness in China, the CFPS questionnaire is selected as the data source. Based on the question “How happy do you feel about your life?” in the questionnaire, we can obtain respondents’ self-rated scores of well-being, which are integer values ranging from 0 to 10. A higher score indicates a higher level of well-being.

Explanatory Variable: The core explanatory variable in this study is educational inequality. Following the approach proposed by Thomas et al. and widely used in academia, the education Gini coefficient for each province is calculated based on the average years of education. A higher education Gini coefficient indicates greater educational inequality. This method is currently the most widely used in academic research and, compared to other measurement methods, the education Gini coefficient calculated using the average years of education better reflects the level of educational equality in a particular region (Senadza, 2012). In Equation 4, μ represents the average years of education, $y_{i}$ and $y_{j}$ represents different levels of education, $p_{i}$ and $p_{j}$ represents the corresponding population proportion for each level of education. 𝑛 is the total number of groups. Specifically, education levels are categorized into five groups: no schooling, primary school or junior high school, senior high school or technical school, junior college, and above. These groups correspond to educational years of 0, 6, 9, 12, and 16 years respectively, based on China’s educational system. Additionally, to minimize estimation bias caused by endogeneity issues, this study matches the lagged one-period education Gini coefficient with the CFPS database.


(4) $$G^{edu}=\frac{1}{\mu }\overset{i-1}{\underset{i=2}{\sum }}\overset{i-1}{\underset{j=1}{\sum }}p_{i}|y_{i}-y_{j}|p_{j}$$

Control variables: drawing on the current research in the field of happiness economics (Ferrer-i-Carbonell and Ramos, 2021; Cuñado and De Gracia, 2012), this study selects control variables from both micro and macro dimensions. At the micro level, control variables mainly consist of individual characteristics of residents, including gender, age, household registration status, relative income, educational level, employment status, marital status, health, religious beliefs, and social trust. At the macro level, control variables include provincial-level economic indicators, such as GDP growth rate and the proportion of the tertiary industry. Data for these variables are sourced from the “China Statistical Yearbook.” Considering that middle-aged individuals may experience reduced well-being due to greater work and family pressures compared to younger and older individuals, the model includes the square term of age (Age^2^) to control for the nonlinear effect of age on well-being. Additionally, relative income is used as a control variable in the model because several studies have shown that factors determining the level of well-being among residents are not absolute income but relative income (Wang et al., 2019).

The micro-level survey data used in this study are derived from the China Family Panel Studies (CFPS) conducted by the China Social Survey Center of Peking University. This dataset is updated every two years and currently covers 31 provinces, municipalities, and autonomous regions nationwide from 2010 to 2018. However, considering that the categorization of well-being levels in 2010 differs from other years, and there is substantial missing data for well-being in 2012 and 2016, this study opts to merge the data from the 2014 and 2018 waves of the Adult Dataset into panel data. After completing data cleaning tasks such as removing samples with missing variables, refusal to answer, or answering “do not know,” a total of 38,456 observations are obtained. The definitions and descriptive statistics of the main variables used in the model are presented in Table 1. From Table 1, it can be observed that the mean value of well-being among Chinese residents is 7.489, indicating a generally high level of well-being. The mean value of education inequality is 0.199, with a maximum value of 0.414. This suggests that the issue of education inequality in China has been alleviated to some extent (see Table 2).

**Table 1:** Descriptive statistics of variables.

Variable	Variable definitions	Mean	Std. dev.	Min	Max
Panel A. Dependent variable					
Happiness	“How happy are you?” Values range from 1 to 10, where 1 indicates “Not happy” and 10 indicates “Extremely happy.”	7.489	2.184	0	10
Panel B. core Explanatory Variable					
$\mathrm{Gin}i^{edu}$	Education Gini Coefficient	0.199	0.257	0.161	0.414
Panel C. Individual characteristics variables					
Gender	Dummy Variable, Male = 1, Other = 0	0.503	0.499	0	1
Age	Resident’s Age	49.470	14.594	16	96
Hukou	Dummy Variable, Urban Household Registration = 1, Other = 0	0.272	0.445	0	1
R income	How would you rate your personal income locally? Values range from 1 to 5, where 1 = Very low and 5 = Very high	2.741	1.051	1	5
Edu	Dummy Variables, 1 = Illiterate, 2 = Primary School, 3 = Junior High School, 4 = High School, 5 = Junior College, 6 = Bachelor’s Degree, 7 = Master’s Degree, 8 = Doctorate	2.571	1.330	1	7
Employ	Dummy Variable, Employed = 1, Other = 0	0.781	0.414	0	1
Marry	Dummy Variable, Married = 1, Other = 0	0.874	0.332	0	1
Health	How would you rate your health condition? Values between 1–5, where 1 = Very healthy, 5 = Unhealthy	3.059	1.227	1	5
Religion	Dummy variable, belief = 1, otherwise = 0	0.157	0.364	0	1
Trust	Degree of trust in strangers: A value between 1 and 10, where 1 = very distrustful and 10 = very trusting	2.030	2.145	0	10
Panel D. Provincial economic variables					
Growth	The GDP growth rate of each province and municipality in the same year (%)	7.411	1.384	3.600	10.900
Proportion	The proportion of the tertiary industry in each province and municipality in the same year (%)	47.656	8.227	35.400	81.000

**Table 2:** Education inequality and residents’ happiness.

	Estimation coefficients	
	Happiness	Happiness
$\mathrm{Gin}i^{edu}$	−4.097*** (0.194)	−2.171*** (0.098)
$\mathrm{Gin}i^{edu}$ ^2	−0.101 (0.254)	−0.081 (0.073)
Gender		−0.102*** (0.009)
Age		−0.001*** (0.002)
Age^2^		0.000*** (0.000)
Hukou		0.103*** (0.013)
R income		0.182*** (0.005)
Edu		0.014*** (0.002)
Employ		−0.058*** (0.009)
Marry		−0.172*** (0.006)
Health		0.023 (0.011)
Religion		0.002 (0.003)
Trust		−0.053*** (0.005)
Growth		−0.006*** (0.001)
Proportion		−2.621*** (0.278)
Province fixed effects		Yes
Year fixed effects		Yes
*N*		38,456

Standard errors in parentheses. *, **, and *** denote significance at the 10, 5, and 1% levels, respectively. Coefficients in the first column of the table represent the regression coefficients of the ordered probit model.

## 4 The empirical results analysis

### 4.1 Baseline regression

In the baseline regression section, this paper will present the estimated coefficients and marginal effects of the ordered probit model. According to the results in the first column of the table, at the 1% significance level, the coefficient of education inequality is significantly negative. The results in the second column show that even after adding a series of control variables and controlling for time and regional fixed effects, the coefficient of education inequality remains significantly negative at the 1% significance level. Additionally, the absolute value of the regression coefficient decreases, indicating a significant negative relationship between education inequality and happiness. This indicates a significant negative impact of education inequality on happiness, particularly in substantially reducing the probability of residents feeling “very happy,” thus confirming the validity of hypothesis 1. The expansion of education inequality undermines social fairness, increases the difficulty for disadvantaged groups to upward mobility through education, and reduces happiness. When disadvantaged groups constitute the majority, the average happiness of society will decrease.

In addition, we added a quadratic term about educational inequality in the article to explore whether it has an inverted U-shaped relationship with happiness. The results were not significant, indicating that there is no non-linear relationship between educational inequality and happiness.

The regression results of the control variables show that in China, the happiness of males is significantly lower than that of females. Age shows a U-shaped relationship with happiness, indicating that the happiness of middle-aged individuals is significantly lower than that of younger and older individuals. The coefficient of household registration is significantly positive, indicating that having an urban Hukou significantly increases the probability of residents feeling happy. The coefficient of relative income is significantly positive, indicating that relative income helps individuals achieve a sense of accomplishment and satisfaction in comparison with others, thereby enhancing happiness. The coefficient of education level is significantly positive, suggesting that an increase in education level contributes to increasing the probability of residents feeling happy. The coefficient of employment status is significantly negative, which may be because employed individuals experience higher work pressure, leading to a decrease in happiness. The marginal effect of marital status on happiness is significantly positive, indicating that being married increases the probability of residents feeling happy compared to other marital statuses. The coefficient of health is significantly negative, suggesting a positive relationship between physical health and happiness. The coefficient of religious belief is significantly positive, indicating that individuals with religious beliefs tend to have higher levels of happiness. This is because religious beliefs can provide spiritual solace and support during hardships and setbacks. The estimated coefficient of social trust is significantly negative, but the marginal effect on happiness is not significant. The coefficients of GDP growth rate and the proportion of the tertiary industry are both significantly negative, suggesting that economic growth does not necessarily lead to an increase in residents’ happiness, thus falling into the “Easterlin paradox.” The impact of these control variables on residents’ happiness aligns with existing research findings.

### 4.2 Heterogeneity analysis

To further investigate whether the effect of educational inequality on happiness varies across different groups, this section uses three dummy variables—happiness level, urban–rural status, and region—as grouping criteria, and then employs the baseline regression model for testing.

#### 4.2.1 Classifying residents by their level of happiness

This study analyzes the impact of educational inequality on well-being through quantile regression, revealing the heterogeneity of this effect across different groups. Compared to conventional OLS regression, quantile regression can capture the differences in well-being responses to educational inequality across different quantile groups, as shown in Table 3.

**Table 3:** Heterogeneity analysis: classified by happiness level.

	25	50	75
	(1)	(2)	(3)
	Happiness	Happiness	Happiness
*Gini* ^edu^	−2.894***	−0.091***	−0.001
	−0.007	−0.019	−0.024
Control variables	Yes	Yes	Yes
Province fixed effects	Yes	Yes	Yes
Year fixed effects	Yes	Yes	Yes
*N*	13,973	12,483	12,000

First, the low well-being group (the 25th percentile) exhibits a significant negative response to educational inequality. This group is likely to face higher economic pressures and limited access to educational opportunities. Educational inequality exacerbates the gap in resource access, leading to further limitations in their social status and opportunities, thus reducing their well-being. This group appears to be more sensitive to educational inequality, likely because the disparity in educational opportunities is more direct and pressing for them, which significantly suppresses their well-being. As a result, the intensification of educational inequality tends to further degrade the quality of life for the low well-being group, making them more sensitive to their perceptions and reactions to inequality.

Next, the middle well-being group (the 50th percentile) shows a relatively smaller response to educational inequality. While educational inequality still exerts some negative impact on them, this group typically enjoys a more stable socio-economic status, with relatively balanced educational opportunities and social resources. Therefore, their well-being may not be as significantly affected as that of the low well-being group. In this case, the impact of educational inequality on the middle well-being group is relatively mild, suggesting that this group is less sensitive to the allocation of educational resources or can compensate for the inequality through other means.

Finally, the high well-being group (the 75th percentile) shows the least response to educational inequality, with coefficients approaching zero. This can be understood from several perspectives: high well-being individuals typically enjoy higher socio-economic status, better access to educational resources, and stronger social support systems. They are likely to have a greater ability to adapt and cope with educational inequality. Given that their resources and opportunities are relatively abundant, the impact of educational inequality on their well-being is small. In other words, the influence of educational inequality on the high well-being group is limited, mainly because they do not rely as much on public education resources, and the determinants of their well-being are more influenced by other social and economic factors.

#### 4.2.2 Classification by urban and rural areas

Considering the significant inequality between urban and rural areas in China, it is possible that urban residents and rural residents perceive educational inequality differently. Therefore, this study classifies the total sample according to urban and rural areas, comparing the differences in the impact of educational inequality on the subjective well-being of urban and rural residents. Table 4 reports the results of grouped regression. Comparing the results between the (1st) column and the (3rd) column, as well as the (2nd) column and the (4th) column, it is found that in different samples, educational inequality is significantly negatively associated with subjective well-being at the 1% significance level. However, the absolute value of the regression coefficient in the rural sample is larger, indicating that rural residents are the primary bearers of the consequences of educational inequality. The reason for this may be that, compared to urban areas, rural areas in China have a relatively lower level of economic development and generally lower levels of education. Therefore, educational inequality not only reduces happiness by widening the urban–rural gap but also decreases happiness by hindering economic development in rural areas.

**Table 4:** Heterogeneity analysis: classified by urban and rural areas.

	Urban	Rural
	(2)	(4)
	Happiness	Happiness
Gini^edu^	−0.190*** (0.103)	−2.316*** (0.003)
Control variables	Yes	Yes
Province fixed effects	Yes	Yes
Year fixed effects	Yes	Yes
*N*	18,253	20,203

#### 4.2.3 Classification by region

Similarly, considering the significant disparities between different regions in China, this study divides the total sample into Eastern and Central-Western regions by region to examine whether the subjective well-being of residents in different regions is affected differently by educational inequality. Table 5 reports the regression results for different groups. The results indicate that educational inequality has little impact on the subjective well-being of residents in the eastern region, but has a significant negative impact on the subjective welfare of residents in the central and western regions. This may be because the Eastern region belongs to economically developed areas, where the government invests heavily in education and the public’s attitude towards education investment is more positive.

**Table 5:** Heterogeneity analysis: classified by regional categories.

	Eastern	Central-western
	(1)	(3)
	Happiness	Happiness
$\mathrm{Gin}i^{edu}$	−0.063*** (0.016)	−2.355*** (0.009)
Control variables	Yes	Yes
Province fixed effects	Yes	Yes
Year fixed effects	Yes	Yes
*N*	16,462	21,994

## 5 Mechanism analysis

The preceding sections have validated the significant negative impact of educational inequality on subjective well-being and its heterogeneity. In this section, starting from the perspectives of income distribution and economic growth, we further explore how educational inequality affects residents’ subjective well-being.

To examine whether educational inequality affects residents’ subjective well-being through income distribution, this study first calculates the level of income inequality in each province and municipality. Specifically, this study adopts the formula for calculating the Gini coefficient, and refers to the income grouping standards in the ‘China Statistical Yearbook.’ After calculating the Gini coefficient for urban and rural residents in each province and municipality separately, the overall Gini coefficient is then calculated using the modified urban–rural weighted method (Bi et al., 2016; Stevenson and Wolfers, 2013). Since income inequality is a continuous variable, when examining the impact of educational inequality on income inequality, OLS regression is used. The results in the (1st) column of Table 6 show that the regression coefficient of educational inequality is significantly positive, indicating that an increase in the degree of educational inequality leads to an expansion of income disparities. The results in the (2nd) column show that the coefficient of income inequality is significantly negative, indicating that an increase in the level of income inequality significantly reduces residents’ subjective well-being. Therefore, it can be inferred that educational inequality reduces subjective well-being by widening income inequality, thus verifying the income distribution effect and confirming Hypothesis 2.

**Table 6:** Mechanism test results.

	(1)	(2)	(3)	(4)
	$\mathrm{Gin}i^{\mathrm{income}}$	Happiness	Lnpgdp	Happiness
$\mathrm{Gin}i^{edu}$	0.574*** (0.027)	−0.206*** (0.021)	−1.106*** (0.025)	−0.861*** (0.005)
$\mathrm{Gin}i^{\mathrm{income}}$		−0.142*** (0.018)		
Lnpgdp				0.071*** (0.003)
Control variables	Yes	Yes	Yes	Yes
Province fixed effects	Yes	Yes	Yes	Yes
Year fixed effects	Yes	Yes	Yes	Yes
*N*	38,456	38,456	38,456	38,456

Similarly, in order to examine whether educational inequality affects residents’ subjective well-being through economic growth, in addition to the income distribution effect, this study also selected the natural logarithm of *per capita* GDP as a proxy variable for economic growth. According to the results in columns (3) and (4) of Table 6, at a significance level of 1%, an increase in educational inequality is not conducive to economic growth, thereby affecting residents’ subjective well-being. Confirmed hypothesis 3. This discovery is consistent with the conclusions of several scholars, including Yang et al. (2020).

## 6 Robustness analysis

To ensure the robustness of the empirical results, this study further conducts tests using alternative models and alternative dependent variables. The results are presented in Table 7.

**Table 7:** Robustness test results.

	(1)	(2)	(3)
	Happiness (OLS)	Happiness (logit)	Happiness
$\mathrm{Gin}i^{edu}$	−5.463*** (0.601)	−0.760*** (0.084)	−0.599*** (0.109)
Control variables	Yes	Yes	Yes
Province fixed effects	Yes	Yes	Yes
Year fixed effects	Yes	Yes	Yes
*N*	38,456	38,456	38,456

### 6.1 Replacement models

In happiness studies, although most scholars advocate treating the happiness data obtained from survey questionnaires as ordinal variables and using ordered probit or ordered logit models for regression. However, Becchetti have pointed out after their research that even if happiness is treated as a continuous variable, it would not substantially affect the empirical results as long as the regression equation’s correctness is ensured (Becchetti et al., 2011). Therefore, in this study, the regression results using the OLS model and the ordered logit model are used as part of the robustness test. As shown in columns (1) and (2) of Table 7, even when the regression model is changed, the coefficient of educational inequality remains significantly negative, indicating the robustness of the baseline regression results.

### 6.2 Replacement of dependent variables

To further ensure the robustness of the empirical results, this study also employs the method of replacing variables. Specifically, a new dummy variable *Happiness* is generated. If the value of the variable *Happiness* is greater than 5, then *Happiness* is set to 1; otherwise, it is set to 0. This converts happiness from an ordinal variable to a binary variable, and a binary probit model is constructed for regression. From column (3) of Table 7, it can be observed that the coefficient of educational inequality is significant at the 1% level, indicating a robust negative relationship between educational inequality and happiness.

## 7 Discussion on endogeneity

Due to the potential issue of omitted variable bias, endogeneity may arise in the model of this study. For example, unobservable variables such as the educational and cultural foundations of different regions, as well as the emphasis placed on education, may simultaneously influence individual well-being and the level of educational inequality, leading to endogeneity problems. To address this issue, this study employs an instrumental variable approach (see Table 8).

**Table 8:** Discussion on endogeneity.

	(1)	(2)
	$EDU^{GOV}$	Happiness
$\mathrm{Gin}i^{edu}$	−0.677*** (0.127)	
EDU^GOV^		−1.112*** (0.128)
Lnpgdp		
Control variables	Yes	Yes
Province fixed effects	Yes	Yes
Year fixed effects	Yes	Yes
*N*	38,456	38,456

Specifically, we use the average proportion of education-related texts in the government work reports of each province from 2014 to 2018 (denoted as $EDU^{GOV}$ ) to measure the provincial government’s level of emphasis on education. The reason for choosing this variable is that government work reports not only reflect the achievements of past government work but also highlight the priorities for future work. Therefore, the proportion of education-related content can be seen as an important indicator of the government’s commitment and focus on education. When discussing the validity of this variable, we need to focus on its relevance and exogeneity.

First, there is a significant correlation between $EDU^{GOV}$ and educational inequality, as government policy orientation and investment directly impact the allocation of educational resources and the fairness of educational opportunities. Second, this variable is exogenous because it comes from government reports, reflecting the government’s stance and priorities on education rather than being a direct result of individual well-being or educational inequality. This characteristic allows $EDU^{GOV}$ to effectively serve as an instrumental variable to help resolve the endogeneity problem in the model, thus enhancing the reliability of the results.

### 7.1 Replacement of the core explanatory variable

To test the robustness of the research findings, we further replace the core explanatory variable from educational inequality (education Gini coefficient) to the Human Opportunity Index (HOI) for education. This modification aims to more comprehensively reflect the impact of educational opportunities on well-being and to overcome the simplification issues that may arise from the education Gini coefficient.

First, we calculate the Human Opportunity Index for education based on higher education enrollment rates across provinces. HOI better captures the multidimensional nature of education, particularly in terms of educational quality and resource distribution. Therefore, we expect that the introduction of the Human Opportunity Index will provide more accurate results and reveal the true impact of educational opportunities on well-being. The results, as shown in Table 9, indicate a negative impact of educational inequality on well-being.

**Table 9:** To replace core explanatory variables.

	(1)
	Happiness
$HOI^{edu}$	−1.063*** (0.016)
Control variables	Yes
Province fixed effects	Yes
Year fixed effects	Yes
*N*	38,456

## 8 Conclusion

This study uses data from the China Family Panel Studies (CFPS) to examine the impact of educational inequality on subjective well-being. The baseline results indicate that educational inequality significantly reduces the probability of individuals feeling happy, and the robustness checks support this conclusion. Heterogeneity analysis shows that the impact of educational inequality is more pronounced for individuals with lower well-being. Additionally, the effect of educational inequality on subjective well-being varies across urban and rural areas, as well as regions. In economically underdeveloped rural areas and central and western regions, educational inequality significantly lowers residents’ well-being, whereas the effect is minimal in urban and eastern regions. Further analysis reveals that educational inequality has significant effects on income distribution and economic growth. Educational inequality affects subjective well-being by exacerbating income inequality and suppressing the level of economic development.

Firstly, optimizing the distribution of educational resources and promoting the establishment of a fair education system is crucial for improving residents’ well-being. Educational inequality not only has a significant negative impact on individual subjective well-being but also hinders the achievement of social equity and sustainable development goals. Therefore, the government should further increase financial investment in education, particularly by creating more opportunities for continuing education, improving the quality of the teaching workforce, and accelerating the construction of a high-quality education system. In this process, policy transparency and accountability mechanisms are essential to ensure that the institutional rules for educational equity are effectively implemented.

Secondly, when formulating education policies, the differences in regional economic development stages should be fully considered. The empirical analysis in this study shows that educational inequality has a significantly heterogeneous impact on different regions and groups at various stages of economic development, particularly in economically underdeveloped rural areas and the central and western regions, where educational inequality significantly reduces residents’ happiness. Therefore, in addition to expanding education coverage, policies should focus on the structural allocation of educational resources, particularly by increasing investment in rural and remote areas to narrow the educational gap between regions. This approach will help improve the overall well-being of society and address the challenges posed by the “Easterlin paradox”.

Although this study has made some progress in exploring the relationship between educational inequality and subjective well-being, there are still some limitations that future research can address. First, this study only uses the educational Gini coefficient as a measure of overall educational inequality. Future research can further decompose educational inequality using the “endowment-effort” binary framework to examine its impact on subjective well-being from multiple dimensions. Such a multidimensional analysis can provide deeper insights into which aspects of educational inequality have the most significant effect on well-being.

Secondly, this study primarily investigates the intrinsic mechanisms of how educational inequality affects subjective well-being from the perspectives of income distribution and economic growth. Future research can consider other potential mechanisms, such as social capital, mental health, and social security, to further reveal how educational inequality influences residents’ well-being through various socio-economic pathways. This would offer a more comprehensive perspective on the multi-layered impact of educational inequality.

## Data Availability

The original contributions presented in the study are included in the article/supplementary material, further inquiries can be directed to the corresponding authors.

## References

[ref1] Aghion P. Caroli E. García-Peñalosa C. (1999). Inequality and economic growth: the perspective of the new growth theories. J. Econ. Lit. 37, 1615–1660. doi: 10.1257/jel.37.4.1615

[ref2] Ahmad M. (2017). Economic freedom and income inequality: does political regime matter? Economies 5:18. doi: 10.3390/economies5020018

[ref3] Becchetti L. Trovato G. Londono Bedoya D. A. (2011). Income, relational goods and happiness. Appl. Econ. 43, 273–290. doi: 10.1080/00036840802570439

[ref4] Bi Y. Lv J. Ma W. (2016). An exploration of the methods to calculate the Gini coefficient in H Province. J. Comput. Theor. Nanosci. 13, 6320–6330. doi: 10.1166/jctn.2016.5564

[ref5] Boehm J. K. Chen Y. Williams D. R. Ryff C. Kubzansky L. D. (2015). Unequally distributed psychological assets: are there social disparities in optimism, life satisfaction, and positive affect? PLoS One 10:e0118066. doi: 10.1371/journal.pone.0118066, PMID: 25671665 PMC4324648

[ref6] Checchi D. García-Peñalosa C. (2008). Labour market institutions and income inequality. Econ. Policy 23, 601–649. doi: 10.1111/j.1468-0327.2008.00209.x

[ref7] Clark A. E. Frijters P. Shields M. A. (2008). Relative income, happiness, and utility: an explanation for the Easterlin paradox and other puzzles. J. Econ. Lit. 46, 95–144. doi: 10.1257/jel.46.1.95

[ref8] Collins R. L. (1996). For better or worse: the impact of upward social comparison on self-evaluations. Psychol. Bull. 119, 51–69. doi: 10.1037/0033-2909.119.1.51

[ref9] Cuñado J. De Gracia F. P. (2012). Does education affect happiness? Evidence for Spain. Soc. Indic. Res. 108, 185–196. doi: 10.1007/s11205-011-9874-x

[ref10] Diener E. Seligman M. E. P. (2004). Beyond money: toward an economy of well-being. Psychol. Sci. Public Interest 5, 1–31. doi: 10.1111/j.0963-7214.2004.00501001.x 26158992

[ref11] Emmons R. A. (2003). “Personal goals, life meaning, and virtue: wellsprings of a positive life” in Flourishing: Positive psychology and the life well-lived. eds. Keyes C. L. M. Haidt J. (Washington: American Psychological Association), 105–128. isbn:978-1-55798-930-7

[ref12] Ferrer-i-Carbonell A. Ramos X. (2021). “Inequality and happiness” in Handbook of labor, human resources and population economics. ed. Zimmermann K. F. (Cham: Springer International Publishing), 1–17. isbn:ISBN 978-3-319-57365-6

[ref13] Graham C. Eggers A. Sukhtankar S. (2004). “Does happiness pay?” in Challenges for quality of life in the contemporary world. eds. Glatzer W. Von Below S. Stoffregen M., vol. 24 (Dordrecht: Social Indicators Research Series, Springer Netherlands), 179–204. isbn:978-90-481-6741-8

[ref14] Hoch S. J. Loewenstein G. F. (1991). Time-inconsistent preferences and consumer self-control. J. Consum. Res. 17, 492–507. doi: 10.1086/208573

[ref15] Kanbur R. Zhang X. (2005). Fifty years of regional inequality in China: a journey through central planning, reform, and openness. Rev. Dev. Econ. 9, 87–106. doi: 10.1111/j.1467-9361.2005.00265.x

[ref16] Mahendru M. Sharma G. D. Hawkins M. (2020). Toward a new conceptualization of financial well-being. J. Public Aff. 22:e2505. doi: 10.1002/pa.2505

[ref17] Mincer J. (1958). Investment in Human Capital and Personal Income Distribution. J. Polit. Econ. 66, 281–302. doi: 10.1086/258055

[ref18] Moore D. Aweiss S. (2003). Outcome expectations in prolonged conflicts: perceptions of sense of control and relative deprivation. Sociol. Inq. 73, 190–211. doi: 10.1111/1475-682X.00052

[ref19] Nadler J. Day M. V. Beshai S. Mishra S. (2020). The relative deprivation trap: how feeling deprived relates to symptoms of generalized anxiety disorder. J. Soc. Clin. Psychol. 39, 897–922. doi: 10.1521/jscp.2020.39.10.897

[ref20] Nelson R. R. (1959). The simple economics of basic scientific research. J. Polit. Econ. 67, 297–306. doi: 10.1086/258177

[ref21] Nomaguchi K. Milkie M. A. (2020). Parenthood and well-being: a decade in review. J. Marriage Fam. 82, 198–223. doi: 10.1111/jomf.12646, PMID: 32606480 PMC7326370

[ref22] Osborne D. García-Sánchez E. Sibley C. G. (2019). “Identifying the psychological mechanisms underlying the effects of inequality on society: the macro-Micro model of inequality and relative deprivation (MIRED)” in The social psychology of inequality. eds. Jetten J. Peters K. (Cham: Springer International Publishing), 249–266. isbn:978-3-030-28855-6

[ref23] Popkova E. G. Chechina O. S. Abramov S. A. (2015). Problem of the human capital quality reducing in conditions of educational unification. MJSS 6, 95–100. doi: 10.5901/mjss.2015.v6n3s6p95

[ref24] Russ M. Bansal G. Parrillo A. (2017). The “Knowledge City” and the “experience City”: the Main, mediating, and moderating effects of education on income and economic inequality. J. Knowl. Econ. 8, 804–829. doi: 10.1007/s13132-015-0278-z

[ref25] Sekreter G. (2019). Emotional intelligence as a vital Indicator of teacher effectiveness. IJSSES 5:286. doi: 10.23918/ijsses.v5i3p286

[ref26] Senadza B. (2012). Education inequality in Ghana: gender and spatial dimensions. J. Econ. Stud. 39, 724–739. doi: 10.1108/01443581211274647

[ref27] Shahbaz M. (2010). Income inequality-economic growth and non-linearity: a case of Pakistan. Int. J. Soc. Econ. 37, 613–636. doi: 10.1108/03068291011060652

[ref28] Shin J. (2018). Relative deprivation, satisfying rationality, and support for redistribution. Soc. Indic. Res. 140, 35–56. doi: 10.1007/s11205-017-1769-z

[ref29] Sima V. Gheorghe I. G. Subić J. Nancu D. (2020). Influences of the industry 4.0 revolution on the human capital development and consumer behavior: a systematic review. Sustain. For. 12:4035. doi: 10.3390/su12104035

[ref30] Slag M. Burger M. J. Veenhoven R. (2019). Did the Easterlin paradox apply in South Korea between 1980 and 2015? A case study. Int. Rev. Econ. 66, 325–351. doi: 10.1007/s12232-019-00325-w

[ref31] Šlaus I. Jacobs G. (2011). Human Capital and Sustainability. Sustain. For. 3, 97–154. doi: 10.3390/su3010097

[ref32] Stevenson B. Wolfers J. (2013). Subjective well-being and income: is there any evidence of satiation? Am. Econ. Rev. 103, 598–604. doi: 10.1257/aer.103.3.598

[ref33] Storper M. Scott A. J. (2008). Rethinking human capital, creativity and urban growth. J. Econ. Geogr. 9, 147–167. doi: 10.1093/jeg/lbn052

[ref34] Tachibanaki T. (2016). “Advances in happiness research: a comparative perspective” in Creative Economy (Japan, Tokyo: Springer). isbn:978-4-431-55752-4

[ref35] Tao C. Xu J. Tao R. Wang Z. Li J. (2022). Influences of relative deprivation on health inequality of rural residents in China: a moderated mediation model. Front. Psychol. 13:1082081. doi: 10.3389/fpsyg.2022.1082081, PMID: 36600700 PMC9807080

[ref36] Wang M. Pang S. Hmani I. Hmani I. Li C. He Z. (2021). Towards sustainable development: how does technological innovation drive the increase in green Total factor productivity? Sustain. Dev. 29, 217–227. doi: 10.1002/sd.2142

[ref37] Wang J. Yan W. Zhang J. (2019). Relative income and subjective well-being of urban residents in China. J. Fam. Econ. Iss. 40, 673–680. doi: 10.1007/s10834-019-09636-0, PMID: 33223788 PMC7677900

[ref38] Yang X. Geng L. Zhou K. (2020). Environmental pollution, income growth, and subjective well-being: regional and individual evidence from China. Environ. Sci. Pollut. Res. 27, 34211–34222. doi: 10.1007/s11356-020-09678-0, PMID: 32542570

[ref39] Yu Z. Wang F. (2017). Income inequality and happiness: an inverted U-shaped curve. Front. Psychol. 8:2052. doi: 10.3389/fpsyg.2017.02052, PMID: 29225588 PMC5705943

[ref40] Zhang Y. (2017). “Secondary education” in Handbook of education in China. eds. Morgan W. J. Gu Q. Li F. (Edward Elgar Publishing), 118–139.

[ref41] Zhang L. Ma Y. (2023). A study of the impact of project-based learning on student learning effects: a Meta-analysis study. Front. Psychol. 14:1202728. doi: 10.3389/fpsyg.2023.1202728, PMID: 37564309 PMC10411581

